# Supplementary material for: HIVprotI: an integrated web based platform for prediction and design of HIV proteins inhibitors
Source: J Cheminform. 2018 Mar 9;10:12. doi: 10.1186/s13321-018-0266-y (PMC5845081; doi:10.1186/s13321-018-0266-y)
Supplement: Supplementary file 2 — Additional file 2. Source code of HIVProtI web server. [file 13321_2018_266_MOESM2_ESM.zip › HIVprotI_Source-code/hivprotI/weka-3-6-6/doc/weka/filters/supervised/instance/SpreadSubsample.html]

SpreadSubsample


---


|  |  |  |
| --- | --- | --- |
| **Overview** \| **Package** \| **Class** \| **Tree** \| **Deprecated** \| **Index** \| **Help** \| **Weka's home** | |  |
| **PREV CLASS**   **NEXT CLASS** | **FRAMES**    **NO FRAMES**     **All Classes** |  |
| SUMMARY: NESTED \| FIELD \| CONSTR \| METHOD | DETAIL: FIELD \| CONSTR \| METHOD |  |


---


## weka.filters.supervised.instance Class SpreadSubsample

```
java.lang.Object
  weka.filters.Filter
      weka.filters.supervised.instance.SpreadSubsample
```

**All Implemented Interfaces:**: java.io.Serializable, CapabilitiesHandler, OptionHandler, RevisionHandler, SupervisedFilter

---

``` public class SpreadSubsample extends Filter implements SupervisedFilter, OptionHandler ```

Produces a random subsample of a dataset. The original dataset must fit entirely in memory. This filter allows you to specify the maximum "spread" between the rarest and most common class. For example, you may specify that there be at most a 2:1 difference in class frequencies. When used in batch mode, subsequent batches are NOT resampled.


Valid options are:

```
 -S <num>
  Specify the random number seed (default 1)
```

```
 -M <num>
  The maximum class distribution spread.
  0 = no maximum spread, 1 = uniform distribution, 10 = allow at most
  a 10:1 ratio between the classes (default 0)
```

```
 -W
  Adjust weights so that total weight per class is maintained.
  Individual instance weighting is not preserved. (default no
  weights adjustment
```

```
 -X <num>
  The maximum count for any class value (default 0 = unlimited).
```

**Version:**
:   $Revision: 5542 $

**Author:**
:   Stuart Inglis (stuart@reeltwo.com)

**See Also:**: Serialized Form

---

**Constructor Summary**

`SpreadSubsample()`


| **Method Summary** | |
| --- | --- |
| `java.lang.String` | `adjustWeightsTipText()`             Returns the tip text for this property |
| `boolean` | `batchFinished()`             Signify that this batch of input to the filter is finished. |
| `java.lang.String` | `distributionSpreadTipText()`             Returns the tip text for this property |
| `boolean` | `getAdjustWeights()`             Returns true if instance weights will be adjusted to maintain total weight per class. |
| `Capabilities` | `getCapabilities()`             Returns the Capabilities of this filter. |
| `double` | `getDistributionSpread()`             Gets the value for the distribution spread |
| `double` | `getMaxCount()`             Gets the value for the max count |
| `java.lang.String[]` | `getOptions()`             Gets the current settings of the filter. |
| `int` | `getRandomSeed()`             Gets the random number seed. |
| `java.lang.String` | `getRevision()`             Returns the revision string. |
| `java.lang.String` | `globalInfo()`             Returns a string describing this filter |
| `boolean` | `input(Instance instance)`             Input an instance for filtering. |
| `java.util.Enumeration` | `listOptions()`             Returns an enumeration describing the available options. |
| `static void` | `main(java.lang.String[] argv)`             Main method for testing this class. |
| `java.lang.String` | `maxCountTipText()`             Returns the tip text for this property |
| `java.lang.String` | `randomSeedTipText()`             Returns the tip text for this property |
| `void` | `setAdjustWeights(boolean newAdjustWeights)`             Sets whether the instance weights will be adjusted to maintain total weight per class. |
| `void` | `setDistributionSpread(double spread)`             Sets the value for the distribution spread |
| `boolean` | `setInputFormat(Instances instanceInfo)`             Sets the format of the input instances. |
| `void` | `setMaxCount(double maxcount)`             Sets the value for the max count |
| `void` | `setOptions(java.lang.String[] options)`             Parses a given list of options. |
| `void` | `setRandomSeed(int newSeed)`             Sets the random number seed. |

**Methods inherited from class weka.filters.Filter**

`batchFilterFile, filterFile, getCapabilities, getOutputFormat, isFirstBatchDone, isNewBatch, isOutputFormatDefined, makeCopies, makeCopy, numPendingOutput, output, outputPeek, toString, useFilter, wekaStaticWrapper`

**Methods inherited from class java.lang.Object**

`equals, getClass, hashCode, notify, notifyAll, wait, wait, wait`

**Constructor Detail**

### SpreadSubsample

```
public SpreadSubsample()
```


**Method Detail**

### globalInfo

```
public java.lang.String globalInfo()
```

:   Returns a string describing this filter

    :   **Returns:**: a description of the filter suitable for displaying in the explorer/experimenter gui

---


### adjustWeightsTipText

```
public java.lang.String adjustWeightsTipText()
```

:   Returns the tip text for this property

    :   **Returns:**: tip text for this property suitable for displaying in the explorer/experimenter gui

---


### getAdjustWeights

```
public boolean getAdjustWeights()
```

:   Returns true if instance weights will be adjusted to maintain
    total weight per class.

    :   **Returns:**: true if instance weights will be adjusted to maintain total weight per class.

---


### setAdjustWeights

```
public void setAdjustWeights(boolean newAdjustWeights)
```

:   Sets whether the instance weights will be adjusted to maintain
    total weight per class.

    :   **Parameters:**: `newAdjustWeights` - whether to adjust weights

---


### listOptions

```
public java.util.Enumeration listOptions()
```

:   Returns an enumeration describing the available options.

    :   **Specified by:**: `listOptions` in interface `OptionHandler`
    :   **Returns:**: an enumeration of all the available options.

---


### setOptions

```
public void setOptions(java.lang.String[] options)
                throws java.lang.Exception
```

:   Parses a given list of options.


    Valid options are:

    ```
     -S <num>
      Specify the random number seed (default 1)
    ```

    ```
     -M <num>
      The maximum class distribution spread.
      0 = no maximum spread, 1 = uniform distribution, 10 = allow at most
      a 10:1 ratio between the classes (default 0)
    ```

    ```
     -W
      Adjust weights so that total weight per class is maintained.
      Individual instance weighting is not preserved. (default no
      weights adjustment
    ```

    ```
     -X <num>
      The maximum count for any class value (default 0 = unlimited).
    ```

    :   **Specified by:**: `setOptions` in interface `OptionHandler`
    :   **Parameters:**: `options` - the list of options as an array of strings **Throws:**: `java.lang.Exception` - if an option is not supported

---


### getOptions

```
public java.lang.String[] getOptions()
```

:   Gets the current settings of the filter.

    :   **Specified by:**: `getOptions` in interface `OptionHandler`
    :   **Returns:**: an array of strings suitable for passing to setOptions

---


### distributionSpreadTipText

```
public java.lang.String distributionSpreadTipText()
```

:   Returns the tip text for this property

    :   **Returns:**: tip text for this property suitable for displaying in the explorer/experimenter gui

---


### setDistributionSpread

```
public void setDistributionSpread(double spread)
```

:   Sets the value for the distribution spread

    :   **Parameters:**: `spread` - the new distribution spread

---


### getDistributionSpread

```
public double getDistributionSpread()
```

:   Gets the value for the distribution spread

    :   **Returns:**: the distribution spread

---


### maxCountTipText

```
public java.lang.String maxCountTipText()
```

:   Returns the tip text for this property

    :   **Returns:**: tip text for this property suitable for displaying in the explorer/experimenter gui

---


### setMaxCount

```
public void setMaxCount(double maxcount)
```

:   Sets the value for the max count

    :   **Parameters:**: `maxcount` - the new max count

---


### getMaxCount

```
public double getMaxCount()
```

:   Gets the value for the max count

    :   **Returns:**: the max count

---


### randomSeedTipText

```
public java.lang.String randomSeedTipText()
```

:   Returns the tip text for this property

    :   **Returns:**: tip text for this property suitable for displaying in the explorer/experimenter gui

---


### getRandomSeed

```
public int getRandomSeed()
```

:   Gets the random number seed.

    :   **Returns:**: the random number seed.

---


### setRandomSeed

```
public void setRandomSeed(int newSeed)
```

:   Sets the random number seed.

    :   **Parameters:**: `newSeed` - the new random number seed.

---


### getCapabilities

```
public Capabilities getCapabilities()
```

:   Returns the Capabilities of this filter.

    :   **Specified by:**: `getCapabilities` in interface `CapabilitiesHandler` **Overrides:**: `getCapabilities` in class `Filter`
    :   **Returns:**: the capabilities of this object **See Also:**: `Capabilities`

---


### setInputFormat

```
public boolean setInputFormat(Instances instanceInfo)
                       throws java.lang.Exception
```

:   Sets the format of the input instances.

    :   **Overrides:**: `setInputFormat` in class `Filter`
    :   **Parameters:**: `instanceInfo` - an Instances object containing the input instance structure (any instances contained in the object are ignored - only the structure is required). **Returns:**: true if the outputFormat may be collected immediately **Throws:**: `UnassignedClassException` - if no class attribute has been set.: `UnsupportedClassTypeException` - if the class attribute is not nominal.: `java.lang.Exception` - if the inputFormat can't be set successfully

---


### input

```
public boolean input(Instance instance)
```

:   Input an instance for filtering. Filter requires all
    training instances be read before producing output.

    :   **Overrides:**: `input` in class `Filter`
    :   **Parameters:**: `instance` - the input instance **Returns:**: true if the filtered instance may now be collected with output(). **Throws:**: `java.lang.IllegalStateException` - if no input structure has been defined

---


### batchFinished

```
public boolean batchFinished()
```

:   Signify that this batch of input to the filter is finished.
    If the filter requires all instances prior to filtering,
    output() may now be called to retrieve the filtered instances.

    :   **Overrides:**: `batchFinished` in class `Filter`
    :   **Returns:**: true if there are instances pending output **Throws:**: `java.lang.IllegalStateException` - if no input structure has been defined

---


### getRevision

```
public java.lang.String getRevision()
```

:   Returns the revision string.

    :   **Specified by:**: `getRevision` in interface `RevisionHandler` **Overrides:**: `getRevision` in class `Filter`
    :   **Returns:**: the revision

---


### main

```
public static void main(java.lang.String[] argv)
```

:   Main method for testing this class.

    :   **Parameters:**: `argv` - should contain arguments to the filter: use -h for help


---


|  |  |  |
| --- | --- | --- |
| **Overview** \| **Package** \| **Class** \| **Tree** \| **Deprecated** \| **Index** \| **Help** \| **Weka's home** | |  |
| **PREV CLASS**   **NEXT CLASS** | **FRAMES**    **NO FRAMES**     **All Classes** |  |
| SUMMARY: NESTED \| FIELD \| CONSTR \| METHOD | DETAIL: FIELD \| CONSTR \| METHOD |  |


---
